# The impact of internet use on adolescents’ health: empirical evidence from China

**DOI:** 10.3389/fpsyt.2024.1404574

**Published:** 2024-05-28

**Authors:** Nianyu Du, Lele Liu, Lianpan Zhang, Shijiu Yin

**Affiliations:** ^1^ School of Economics, Qufu Normal University, Rizhao, China; ^2^ School of Economics and Management, Anhui Agricultural University, Hefei, China

**Keywords:** internet use, adolescents, physical health, mental health, China

## Abstract

**Background:**

With the continuous improvement in economic levels in various countries and the rapid development of the Internet, adolescents’ Internet use has become increasingly frequent. Many studies have explored the relationship between Internet use and adolescent health, but the possible mechanisms involved are unclear, and few have focused on Chinese adolescents.

**Methods:**

Based on the data from the China Family Panel Studies 2010, 2014, 2016, 2018, and 2020, this study used the ordered probit model and OLS model to explore the impact of Internet use on self-rated health and mental health of Chinese adolescents and analyzed the possible mechanisms and heterogeneity involved.

**Results:**

The results revealed that adolescents’ Internet use negatively affected their physical and mental health. Parent-child conflict, academic performance, and self-efficacy were the important mechanisms of internet use affecting adolescents’ health. Moreover, the negative effects of Internet use on adolescents’ physical and mental health were heterogeneous in boarding, mother’s education level, and family per capita income.

**Conclusion:**

Adolescents exhibit a pattern of using the internet that influences their health status. Our finding that internet use may decrease adolescents’ health provides important insights into understanding the relationship between internet use behavior and adolescents’ health and suggests that intervention should be taken on adolescents’ internet use.

## 1 Introduction

Strong young people ensure the strength of the country; therefore, adolescents’ health is crucial to a country’s future. Since the end of the 20th century, adolescents’ health has been the focus of international public health. In 2016, the World Health Organization (WHO) adopted a resolution on the global strategy for the health of women, children, and adolescents^1^. Adolescence is a critical transitional period in the growth and development of children into adulthood. Studies have shown that adolescents’ health impacts their behavior and health in adulthood. For instance, Kendig et al. (1) found childhood health influences later life health. Hetlevik et al. (2) demonstrated that self-rated health in adolescence is related to the risk of having multi-illness in early adulthood. In addition, Machů et al. (3) found an association between adolescent mental health and work-family trajectories in young adulthood. Therefore, it is particularly important to pay attention to adolescent health.

Studies on the impact of Internet use on adolescent health have been conducted worldwide; however, a consensus has not been reached. Some scholars believe that Internet use is beneficial to adolescents’ physical and mental health. The Internet is a source of health-related information (4) and can help adolescents search for health-related information. In addition, the Internet facilitates information transfer, communication, and access to resources (5). Proper Internet use can help adolescents broaden their horizons and relieve stress and fatigue (6). Moreover, Internet use helps expand the size of social networks, providing individuals with social and emotional support (7). Adolescents can maintain social connections through the Internet, deepen intimate relationships with friends (8), and reduce levels of loneliness and depression, thereby improving their mental health (9).

Conversely, some scholars argue that Internet use is detrimental to adolescents’ physical and mental health. These scholars suggest that adolescents lack cognitive skills and practical experience and their self-control ability is still in the stage of improvement (10). Adolescents are more likely to indulge in the Internet and stay online longer than adults (11, 12). Long-term Internet use can occupy adolescents’ time and limit healthy activities, such as outdoor activities and face-to-face interactions with family and friends) as well as hinder sleep, rest, and self-care (13–15). Furthermore, long-term Internet use increases periods of sitting, which often leads to being overweight, obesity, and vision and spinal problems (16). In addition, some studies have suggested that Internet use is associated with anxiety and depression in adolescents (17). Excessive Internet use among adolescents not only does not alleviate loneliness but may even lead to withdrawal from real-life interpersonal relationships, exacerbating loneliness (18). Moreover, excessive Internet use among adolescents may cause mental health problems, including anxiety and depression, and problems with social adaptation (19). Long-term Internet use can also exacerbate anxiety and depression in adolescents (20). Furthermore, smartphones provide convenient Internet access for adolescents, which may create problems in interpersonal relationships, emotional states, and physical health. Studies have found that excessive use of mobile phones to surf the Internet may lead to mental health problems, such as anxiety and unease, affecting real-life emotional experiences (21).

From the above, it can be seen that relevant research mainly focuses on developed countries, and there is insufficient evidence from developing countries. Therefore, this article enriches the relevant literature by exploring the causal relationship between Internet use and adolescent health in China, the largest developing country. In 2021, China had 1.032 billion Internet users, with a national Internet penetration rate of 73.0% (22), Among them, 191 million users were minors^2^, and the Internet penetration rate of minors was 96.8% (23). The Internet has become an integral part of adolescents’ lives, significantly impacting their lifestyles. However, in China, most scholars mainly discuss the impact of Internet use on adult health (24–26).

As mentioned above, the current research has some shortcomings: First, compared with adults, there are fewer studies on the impact of Internet use on adolescent health. Whether, how, and why Internet use affects adolescent health is still unclear. Second, Most of the relevant studies on Chinese adolescents only use cross-sectional data or have a small sample size (27, 28), which is not representative and random enough to ensure the accuracy of the conclusions.

This paper makes three major contributions to the literature. First, we attempted to estimate the causal relationship between Internet use and the health of Chinese adolescents, enriching the relevant evidence from developing countries. Second, we use CFPS 2010–2020 longitudinal data, a nationally representative survey, a large number of data samples, a fixed effect model, and an instrumental variable method to determine the causal relationship between Internet use and adolescents’ physical and mental health. Finally, we explored the mechanism of Internet use affecting adolescent health from the perspective of family and school and conducted heterogeneity analysis from multiple perspectives such as boarding and education level of the mother.

## 2 Hypothesis development

Adolescents’ Internet use may increase parent–child conflict, thereby impacting their physical and mental health. Due to age and cognitive limitations, adolescents are mainly under the supervision of their parents, and the family holds importance for them. Parent–child relationships play an important role in the healthy growth of adolescents. Positive parent–child relationships provide a warm haven for adolescents, ensuring secure attachment and helping adolescents establish a positive self-concept. This reduces the threat of Internet addiction and promotes healthy physical and mental development (29, 30). In contrast, negative parent–child relationships can lead to dysfunctional self-cognition in adolescents, which may cause Internet addiction. This can trigger emotional problems, such as depression and loneliness (31). Furthermore, Internet dependence may exacerbate parent–child conflicts (32).

In addition, adolescents’ Internet use may negatively impact their academic performance, thereby affecting their physical and mental health. Most adolescents are in the schooling stage and face significant academic pressure (33). Studies have shown a strong correlation between adolescents’ Internet use and low academic performance, indicating that using the Internet may hinder academic performance (34, 35). Poor academic performance often places academic pressure on adolescents, increasing their anxiety and loneliness and adversely affecting their mental health (36). Stress can affect an individual’s autonomic nervous system and suppress their immune system (37), suggesting that excessive academic pressure may increase the risk of disease in adolescents.

Moreover, adolescents’ Internet use may reduce their academic self-efficacy, affecting their physical and mental health. Academic self-efficacy refers to an individual’s perception of their ability to achieve predetermined academic goals (38). High academic self-efficacy is associated with a high evaluation of one’s abilities, confidence in dealing with anxiety and other emotions related to examinations, and good mental health (39–41). However, inappropriate Internet use may damage academic self-efficacy, thereby hindering physical and mental health (42, 43). Based on the above content, the following hypotheses were proposed:

H1: Internet use negatively impacts adolescents’ physical and mental health.

H2: The negative impact of Internet use on adolescents’ physical and mental health occurs through increased parent–child conflict, decreased academic performance, and decreased academic self-efficacy.

## 3 Materials and methods

### 3.1 Data and participants

This study used data from the China Family Panel Studies (CFPS) released by the China Social Sciences Research Center of Peking University. The data sample survey was officially launched in 2010 and has been conducted every two years, covering 25 provinces, cities, and autonomous regions. The data had been updated until 2020 with six survey data sets: 2010, 2012, 2014, 2016, 2018, and 2020. The samples for the CFPS were drawn using a multistage probability with implicit stratification (44). The research scope covered 25 provinces/cities/autonomous regions outside Inner Mongolia, Hainan, Tibet, Qinghai, Ningxia, and Xinjiang. The target sample size was 16000 households, and the respondents included all family members in the sample households. The CFPS database tracks and collects information at the individual, household, and community levels, covering a wide range of research topics, including economic activities, family relationships and dynamics, population migration, and health. These data have been widely used in health research (45, 46).

The 2012 CFPS data lacked the key variables of Internet use, so we used the five CFPS survey data in 2010, 2014, 2016, 2018, and 2020. CFPS’ children and adolescents database contained data on self-rated health, mental health, Internet use, etc. This paper used stata17 to make the following adjustments to the data. First, this article excluded adolescents under the age of 10 because they lacked information about Internet use. Secondly, this paper included individuals aged 10–15^3^ and matched the database of adolescents, adults, and family relations to obtain the required variables. Thirdly, to unify the data for each year, this paper only retained 162 districts and counties from the original survey in 2010. Finally, the samples with missing values of key variables were excluded and 7993 final samples were obtained.

### 3.2 Measures

#### 3.2.1 Explained variable

The dependent variables were the adolescents’ self-rated health and mental health. The self-rated health of adolescents was measured using the question “How would you rate your physical health?” Responses were rated on a five-point Likert scale (1 = very healthy; 5 = unhealthy).

The CFPS uses the Kessler Psychological Distress Scale (K6)^4^ and the Center for Epidemiological Studies Depression Scale (CES-D)^5^ to measure mental health. K6 (47) and CES-D (48) are widely used in research to assess psychological well-being (49–51). **Table 1** shows the K6 and CES-D scale questions. The 2010 and 2014 surveys used the same six questions to measure the mental health status of the respondents, whereas the 2012 and 2016 surveys used a different set of 20 questions. In addition, the 2018 and 2020 surveys revised and simplified these 20 questions. However, to be able to link with the data in 2012, the scores of 20 questions in 2018 and 2020 were retained, and we used the scores of 20 questions for research. This paper reverse scoring of mental health problems, so that the higher the score, the worse the mental health. To avoid dimensional disparities and facilitate the interpretation of coefficients, the total scores of K6 and CES-D were standardized (with a mean of 0 and a standard deviation of 1), which allows for a unified measurement of mental health scores across different years after standardization (52).

**Table 1:** K6 and CES-D scale problems.

CSE-D	K6
I am worried about some trivial things.	How often in the past month did you feel so depressed that nothing could cheer you up?
I have a poor appetite and do not want to eat.	How often in the past month did you feel nervous?
I feel depressed despite the help from relatives and friends.	How often in the past month did you feel restless or fidgety?
I find myself not worse than others.	How often in the past month did you feel hopeless?
I cannot concentrate on things.	How often in the past month did you feel that everything was an effort?
I am in a low spirit.	How often in the past month did you feel that life was meaningless?
I find it difficult to do anything.	
I find the future promising.	
I feel that I have been a loser for a long time.	
I feel scared.	
I cannot sleep well.	
I feel happy.	
I talk less than usual.	
I feel lonely.	
I find that people are not friendly to me.	
I have a happy life.	
I cried or I want to cry	
I feel sad.	
I find that others do not like me.	
I feel that I cannot continue with my life.	

In view of the utilization of the Kessler Psychological Distress Scale (K6) and the CES-D scales to construct empirical psychological health data, certain measures were employed to ensure the quality of these scales (**Table 2**). Firstly, a reliability analysis was conducted. Cronbach’s alpha coefficient was computed, which is widely recognized to indicate excellent internal consistency if exceeding 0.8, good if falling between 0.7 and 0.8, and poor if below 0.7 (53). The results revealed Cronbach’s alpha coefficients above 0.7 for all scales, suggesting a favorable level of reliability for the scales used. Secondly, a structural validity analysis was performed through the implementation of the Exploratory Factor Analysis (EFA) technique. Both the KMO measure of sampling adequacy and Bartlett’s Test of Sphericity were utilized. The findings exhibited KMO values surpassing 0.7, and the chi-square statistic derived from Bartlett’s Test of Sphericity was statistically significant, thereby endorsing the scales’ sound structural validity (54, 55).

**Table 2:** reliability and validity analysis.

Scale	K6	CES-D20	CES-D8
Cronbach’s alpha	0.790	0.963	0.714
KMO test value	0.848	0.975	0.774
Bartlett’s Test of Sphericity Chi-Square Statistic	6308.575	85087.993	3182.920
P-value	P<0.001	P<0.001	P<0.001
Observations	4342	1511	2140

#### 3.2.2 Core explanatory variable

The core explanatory variable was adolescents’ Internet use, which was measured using the question “Do you use/Have you ever used the Internet?” If the answer was “yes,” Internet use was assigned a value of 1; otherwise, it was assigned a value of 0.

#### 3.2.3 Mediating variable

We included parent-child conflict, academic performance, and academic self-efficacy as mediating variables. Parent-child conflict was measured using the question “How many times have you and your parents quarreled in the past month?” Academic performance was measured using the question “How do you rate your academic performance?” Responses were rated on a five-point Likert scale (1 = very dissatisfied; 5 = very satisfied). Academic self-efficacy was measured using the question “How good do you think you are as a student?” Responses were rated on a five-point Likert scale (1 = very poor; 5 = excellent).

#### 3.2.4 Control variable

Based on previous studies (28, 46), we controlled for variables that may affect adolescent health, including individual, behavioral, family, and provincial dummy variables. Individual variables included age, gender(male/female, as assigned at birth), ethnicity, residence, academic stage, and whether they were in a single-parent family. Behavioral variables included whether they had social health insurance, and whether they lived on campus. Family variables included the mother’s educational level, number of family members, and per capita income in the past 12 months. To preserve the zero per capita income of teenage households, per capita income was log-transformed after adding 1. Age and household size were continuous variables, whereas the remaining variables were categorical.

### 3.3 Data analyses & procedure

In order to explore the impact of Internet use on Chinese adolescents’ health, we used stata17.0 measurement software and CFPS data from 2010, 2014, 2016, 2018 and 2020 to establish a fixed effect model to determine the causal relationship between Internet use and adolescents’ self-rated health and mental health. The self-rated health data included in this study were discrete ordinal variables, and the mental health data were continuous variables. Therefore, this study used an ordered probit model for self-rated health benchmark regression, and an OLS model for mental health benchmark regression. Ordered probit model as follows:


(1) $$Health_{\mathrm{ijt}}^{*}=\beta_{0}+\beta_{1}Internet_{ijt}+\gamma X_{ijt}+\delta_{t}+\delta_{j}+\varepsilon_{ijt}$$

where i represents individuals; j represents the individual’s province; t represents years; explained variable $Health_{\mathrm{ijt}}^{*}$ represents the underlying variable behind the ordinal categorical variable $Health_{ijt}$ ; $Internet_{ijt}$ represents the core explanatory variable indicating whether the adolescent uses the Internet; $X_{ijt}$ represents other control variables; $\delta_{t}$ is the fixed effect of the year; $\delta_{j}$ is the fixed effect of the province; and $\varepsilon_{ijt}$ is a random error term, which follows a normal distribution. As the true health status $Health_{\mathrm{ijt}}^{*}$ of individual i cannot be directly observed, it was treated as a latent variable. The relationship between latent variable $Health_{\mathrm{ijt}}^{*}$ and health status $Health_{ijt}$ of individual i was expressed as follows:


(2) $$Health_{ijt}=\{\begin{array}{c} 1,\;if\;Health_{\mathrm{ijt}}^{*}\;\leq \;\mu_{1}\\ 2,if\;\mu_{1}<Health_{\mathrm{ijt}}^{*}\;\leq \;\mu_{2}\\ \cdots \\ K,\;if\;\mu_{k-1}\;<Health_{\mathrm{ijt}}^{*}\;\leq \;\mu_{k} \end{array}\}$$

In **Equation (2)** $Health_{\mathrm{ijt}}^{*}$ is a discrete variable with a directly observable range of [1, K], in which K ∈ R; $\mu_{k}$ is a new set of functions; and $\mu_{1}<\mu_{2}<\cdots <\mu_{k}$ . $Health_{\mathrm{ijt}}^{*}$ is divided into K non-overlapping intervals such that the probability of $Health_{ijt}$ taking a specific value k is:


(3) $$P(Health_{ijt}=k)=P(\mu_{k-1}<Health_{\mathrm{ijt}}^{*}\leq \mu_{k})=\text{Φ(}\mu_{k}-W)-\text{Φ(}\mu_{k-1}-W\text{) }$$

where $W=\beta_{0}+\beta_{1}Internet_{ijt}+\gamma X_{ijt}+\delta_{t}+\delta_{j}$ ; Φ(·) is the cumulative density function of the standard normal distribution and k=1,⋯,5 . We focus on the coefficient $\beta_{1}$ , which measures the impact of Internet use on adolescent health. If $\beta_{1}$ is negative, the explanatory variable and probability of lower-level values increase, whereas the probability of higher-level values decreases. OLS model is as follows:


(4) $$Mental\;health_{ijt}=\beta_{0}+\beta_{1}Internet_{ijt}+\gamma X_{ijt}+\delta_{t}+\delta_{j}+\varepsilon_{ijt}$$

where explained variable $Mental\;health_{ijt}$ represents personal mental health, Other variables are the same as in Equation (1) (1).

### 3.4 Ethical considerations

The data was approved by the Biomedical Ethics Committee of Peking University (IRB00001052–14010). In addition, our team has applied for and obtained the right to use the data through the Open Research Data Platform of Peking University.

## 4 Results

### 4.1 Descriptive statistics

The demographic characteristics and descriptive statistics of the variables are shown in **Table 3**. Physical and mental health in the sample was high. The number of teenagers using the Internet accounted for 43.5% of the total sample. The participants generally had more than one quarrel with their parents per month. The participants were generally satisfied with their academic performance and had high academic self-efficacy.

**Table 3:** Meaning and descriptive statistics of main variables.

Variable	Definition	Mean	SD	Min	Max
Explained variable					
Self-rated health	Very healthy = 1,healthy = 2,relatively healthy = 3,average = 4,Unhealthy = 5	1.752	0.882	1	5
Mental health	K6 and CES-D reverse scoring and standardization	0	1	-1.474	5.669
Sick in hospital	Whether hospitalized due to illness in the past year: no = 0, yes = 1	0.137	0.344	0	1
Depressed mood	Frequency of feeling depressed	0	1	-0.770	3.450
Explanatory variable					
Internet use	No = 0, yes = 1	0.435	0.496	0	1
Mediating variable					
Parent–child conflict	Number of quarrels with parents in the past month	0.984	2.600	0	40
Academic performance	Very dissatisfied = 1, dissatisfied = 2, average = 3, satisfied = 4, very satisfied = 5	3.372	0.920	1	5
Academic self-efficacy	Very poor = 1, poor = 2, average = 3, good = 4, excellent = 5	3.181	0.850	1	5
Control variable					
Age	Adolescent’s age	12.47	1.704	10	15
Gender	Woman = 0, man = 1	0.525	0.499	0	1
Ethnicity	Non-Han = 0, Han = 1	0.872	0.334	0	1
Residence	Rural = 0, urban = 1	0.372	0.483	0	1
Current academic stage	Current academic stage of the adolescent^†^	2.416	0.521	1	5
Single parent family	No = 0, yes = 1	0.015	0.121	0	1
Social medical insurance	No = 0, yes = 1	0.423	0.494	0	1
School boarding	No = 0, yes = 1	0.283	0.451	0	1
Mother’s educational level	Mother’s educational level^‡^	2.331	1.163	1	8
Family size	Number of family members of the adolescent	5.061	1.728	1	15
Per capita income	Per capita household income in the past 12 months (ln)	7.090	1.153	0	12.150

^†-^Kindergarten/preschool = 1, primary school = 2, junior high school = 3, high school/secondary school/technical school/vocational high school = 4, junior college = 5.

^‡-^No formal education = 1, primary school = 2, junior high school = 3, high school/secondary technical school/technical school/vocational high school = 4, junior college = 5, bachelor’s degree = 6, master’s degree = 7, doctorate degree = 8.

On average, the adolescents in the sample were 12.47 years old, with slightly more males than females (52.5%). Most of the adolescents were Han Chinese, accounting for 87.2% of the sample. A larger percentage of the sample lived in rural areas (62.8%) compared to cities and towns (37.2%). Most of the adolescents interviewed were in primary and junior high school. Adolescents from single-parent families accounted for only 1.5% of the total sample. About 42.3% of adolescents had social medical insurance. Additionally, 28.3% of adolescents in the sample were boarding in schools. The average education level of adolescent mothers was 2.331, mainly with primary and junior high school education. The average family size was 5.016, and the average per capita income (natural logarithm) of the sample adolescent families in the past year was 7.090, and the overall income level was good.

### 4.2 Baseline results

Based on Equations (3) and (4). **Table 4** presents the benchmark regression results of the impact of Internet use on adolescents’ physical and mental health. We added individual (Columns 1 and 4) and behavioral characteristics (Columns 2 and 5) and controlled for individual, behavioral, and family characteristics (Columns 3 and 6). All results were controlled by provincial fixed effect and year fixed effect. Internet use negatively impacted self-reported and mental health, supporting H1.

**Table 4:** Impact of Internet use on adolescents’ physical and mental health.

Variable	Self-rated health			Mental health		
	(1)	(2)	(3)	(4)	(5)	(6)
Internet use	0.065^**^ (2.170)	0.068^**^ (2.271)	0.067^**^ (2.186)	0.048^*^ (1.874)	0.050^**^ (1.971)	0.086^***^ (3.302)
Age	0.015 (1.236)	0.012 (0.985)	0.013 (1.049)	0.014 (1.314)	0.012 (1.178)	0.006 (0.585)
Gender	-0.050^*^ (-1.934)	-0.051^**^ (-1.962)	-0.049^*^ (-1.878)	-0.019 (-0.856)	-0.019 (-0.861)	-0.013 (-0.582)
Ethnicity	0.020 (0.411)	0.023 (0.453)	0.024 (0.472)	-0.081^*^ (-1.857)	-0.081^*^ (-1.872)	-0.059 (-1.347)
Urban	-0.115^***^ (-3.963)	-0.087^***^ (-2.925)	-0.089^***^ (-2.862)	-0.052^**^ (-2.151)	-0.039 (-1.582)	0.010 (0.380)
Current academic stage	0.033 (0.853)	-0.001 (-0.014)	-0.001 (-0.033)	0.000 (0.003)	-0.014 (-0.387)	0.006 (0.174)
Single parent family	0.236^**^ (1.985)	0.239^**^ (2.013)	0.240^**^ (2.024)	0.084 (0.913)	0.082 (0.891)	0.099 (1.084)
Social medical insurance		0.017 (0.516)	0.016 (0.501)		-0.023 (-0.847)	-0.011 (-0.406)
School boarding		0.120^***^ (3.677)	0.121^***^ (3.695)		0.053^*^ (1.869)	0.044 (1.562)
Mother’s educational level			0.007 (0.508)			-0.042^***^ (-3.681)
Family size			0.004 (0.422)			0.003 (0.319)
Per capita income			-0.002 (-0.132)			-0.055^***^ (-4.247)
Province	Yes^7^	Yes	Yes	Yes	Yes	Yes
Year	Yes	Yes	Yes	Yes	Yes	Yes
Observations	7993	7993	7993	7993	7993	7993

Values in parentheses are z-values (robust standard errors). *, **, and *** indicate p< 0.10, p< 0.05, and p< 0.01, respectively.

^7^ "Yes" indicates that the regression includes the corresponding fixed effects. The same applies to the "yes" in the subsequent tables.

### 4.3 Robustness checks

#### 4.3.1 Endogenous treatment

Due to possible missing variables and reverse causality, endogeneity problems may arise when using regression models for estimation. This study used instrumental variables to alleviate possible endogeneity. Following previous studies (56, 57), we selected the Internet penetration rate in the community where the adolescents lived each year as an instrumental variable^6^ and re-estimated the original model using two-stage least squares (2SLS). An effective instrumental variable should satisfy two conditions: correlation and exogeneity. The Internet penetration rate shows the level of Internet infrastructure in the community and the Internet use of other adolescents, while adolescents’ Internet use is affected by community network infrastructure and community peer effect, which meets the correlation. Furthermore, the Internet penetration rate of community adolescents does not directly show physical and mental health, satisfying the exogeneity condition.

**Table 5** presents the estimation results of the instrumental variable using 2SLS. A weak instrumental variable test was conducted on the instrumental variables in this article. The F-statistic in the first-stage regression results of self-reported health and mental health was larger than 10, indicating that there was no weak instrumental variable problem with the instrumental variables. According to the second-stage regression results, Internet use had a significant negative impact on adolescents’ physical and mental health. The results remained significant after considering endogeneity, consistent with expectations, indicating the robustness of the estimation results.

**Table 5:** Endogenous treatment.

Variable	Self-rated health		Mental health	
	First stage	Second stage	First stage	Second stage
IV	0.339^***^ (18.241)		0.339^***^ (18.241)	
Internet use		0.303^***^ (3.035)		0.385^***^ (3.317)
F statistic	332.73		332.73	
Control variables^8^	Yes	Yes	Yes	Yes
Province	Yes	Yes	Yes	Yes
Year	Yes	Yes	Yes	Yes
Observations	7993	7993	7993	7993

Values in parentheses are z-values (robust standard errors). The reduction in sample size was due to missing variables in villages and towns and the small number of samples in some villages and towns. *** indicates p< 0.01, respectively.

^8^To save space, coefficients of control variables (refer to **Table 3** for details) are no longer reported.

#### 4.3.2 Replacing variable indicators

As adolescents may not be able to accurately evaluate their health status due to limitations in their cognitive abilities and age, the question “Whether hospitalized due to illness in the past year?” was used as a proxy variable for self-reported health to objectively measure the health status of adolescents. A value of 1 was assigned if there was hospitalization due to illness, and a value of 0 was assigned otherwise. Referring to existing literature (52), The question “I feel depressed” in the K6 scale and the question “How often do you feel depressed, depressed and unable to excite yourself” in the CES-D scale were used as alternative variables of mental health. The higher the value, the higher the frequency of depression. The scores of the two questions are standardized (the mean value is 0, and the standard deviation is 1).

As shown in **Table 6**, adolescents’ Internet use increased the frequency of hospitalizations and depression. After replacing the explanatory variable indicator, Internet use hindered adolescents’ physical and mental health, which was consistent with the benchmark regression results.

**Table 6:** Robustness test.

variable	Variable indicators replaced		Replace model		Exclude the new crown epidemic		Excluding extreme values	
	Sick in hospital	Depressed mood	Self-rated health	Mental health	Self-rated health	Mental health	Self-rated health	Mental health
Internet use	0.154^***^ (3.461)	0.153^***^ (5.730)	0.051^**^ (2.323)	0.109^***^ (4.021)	0.056^*^ (1.690)	0.089^***^ (3.321)	0.066^**^ (2.102)	0.083^***^ (3.148)
Control variables	Yes	Yes	Yes	Yes	Yes	Yes	Yes	Yes
Province	Yes	Yes	Yes	Yes	Yes	Yes	Yes	Yes
Year	Yes	Yes	Yes	Yes	Yes	Yes	Yes	Yes
Observations	7993	7993	7993	7993	7114	7114	7624	7624

Values in parentheses are z-values (robust standard errors). *, **, and *** indicate p< 0.10, p< 0.05, and p< 0.01, respectively.

#### 4.3.3 Changing the measurement model

To prevent estimation bias due to model selection, we used a linear regression model for adolescents’ self-rated health and used ordered probit model for adolescents’ mental health. As shown in **Table 6**, Internet use had a significant negative impact on adolescent physical and mental health. The coefficient signs and significance after model substitution were the same as those of the benchmark regression results, indicating that the estimation results were robust.

#### 4.3.4 Excluding the influence of the new crown epidemic

Affected by the closure and control policy of the new crown epidemic, adolescents’ classes and entertainment can only be carried out at home, which has increased the frequency of adolescents’ Internet use. At the same time, the inability to exercise and face-to-face communication with classmates will also have a certain impact on the physical and mental health of adolescents. To reduce the interference with the regression results, the data samples affected by the epidemic in 2020 were deleted and re-estimated. As shown in **Table 6**, the impact of Internet use on physical and mental health remained significantly negative, which was consistent with the benchmark regression results.

#### 4.3.5 Excluding extreme values

To prevent the estimation errors caused by extreme values, we shrank the number of parent-child quarrels, the number of family population, and the per capita income of families by 1% on the left and right sides. As shown in **Table 6**, Internet use had a significant negative impact on adolescent physical and mental health.

### 4.4 Mechanism checks

To analyze the mechanism of the effect of Internet use on adolescents’ physical and mental health, this study regressed parent-child conflict, academic performance, and academic self-efficacy. regression results are shown in **Table 7**. The coefficient of using the Internet in column (1) was significantly positive. The use of the Internet increased the number of quarrels between adolescents and their parents. The coefficient of parent-child conflict in column (2) was significantly positive, and the coefficient of parent-child conflict in column (3) was significantly positive, which indicated that the more arguments with parents, the more likely they were to hurt Adolescents’ physical and mental health. Similarly, academic achievement and academic self-efficacy were beneficial to adolescents’ physical and mental health, while adolescents’ use of the Internet reduced academic achievement and academic self-efficacy, thereby damaging their physical and mental health.

**Table 7:** Mechanism analysis.

Variable	Parent–child conflict	Self-rated health	Mental health	Academic performance	Self-rated health	Mental health	Academic self-efficacy	Self-rated health	Mental health
	(1)	(2)	(3)	(4)	(5)	(6)	(7)	(8)	(9)
Internet use	0.343^***^ (10.287)			-0.097^***^ (-3.431)			-0.068^**^ (-2.455)		
Parent–child conflict		0.023^***^ (4.101)	0.058^***^ (9.575)						
Academic performance					-0.113^***^ (-7.494)	-0.078^***^ (-5.722)			
Academic self-efficacy								-0.131^***^ (-7.890)	-0.096^***^ (-6.649)
Control variables	Yes	Yes	Yes	Yes	Yes	Yes	Yes	Yes	Yes
Province	Yes	Yes	Yes	Yes	Yes	Yes	Yes	Yes	Yes
Year	Yes	Yes	Yes	Yes	Yes	Yes	Yes	Yes	Yes
Observations	7993	7993	7993	7993	7993	7993	7993	7993	7993

The values in parentheses are z-values (robust standard errors). ** and *** indicate p< 0.05 and p< 0.01, respectively.

Thus, Internet use negatively impacted physical and mental health by increasing parent-child conflict and decreasing academic performance and self-efficacy, supporting H2.

### 4.5 Heterogeneity analysis

#### 4.5.1 Heterogeneity analysis of self-rated health impact

**Table 8** reports the impact of Internet use on adolescents’ self-rated health by Boarding, mother’s educational level, and per capita household income. Internet use had a significant negative impact on nonboarding adolescents’ self-rated health but not boarding adolescents’ self-rated health. Adolescents whose mothers’ education level was primary school or below were more likely to damage their self-rated health by using the Internet. Internet use had a significant negative impact on the self-rated health of adolescents from high-income families but not the self-rated health of adolescents from low-income families.

**Table 8:** Heterogeneity analysis: Self-rated health.

Variable	Boarding or not		Mother’s educational level		Per capita household income^9^	
	Non-boarding	Boarding	Primary school and below	Above primary school	Low-income families	High-income families
Internet use	0.073^**^ (1.967)	0.049 (0.856)	0.107^*^ (1.711)	0.058^*^ (1.649)	0.039 (1.146)	0.188^**^ (2.531)
Control variables	Yes	Yes	Yes	Yes	Yes	Yes
Province	Yes	Yes	Yes	Yes	Yes	Yes
Year	Yes	Yes	Yes	Yes	Yes	Yes
Observations	5729	2264	2366	5627	6382	1611

Values in parentheses are z-values (robust standard errors). * and ** indicate p< 0.1 and p< 0.05, respectively.

^9^The per capita income of families is divided according to the 80% quantile of the whole sample, and those higher than the 80% quantile are high-income families, otherwise they are low-income families.

#### 4.5.2 Heterogeneity analysis of mental health effects

**Table 9** reports the impact of Internet use on adolescents’ mental health by Boarding, mother’s educational level, and per capita household income. Internet use had a significant negative impact on nonboarding adolescents’ mental health but not boarding adolescents’ self-rated health. Adolescents with highly educated mothers faced greater psychological pressure when using the Internet. Internet use had a significant negative impact on the mental health of adolescents from low-income families but not the mental health of adolescents from high-income families.

**Table 9:** Heterogeneity analysis: Mental health.

Variable	Boarding or not		Mother’s educational level		Per capita household income	
	Non-boarding	Boarding	Primary school and below	Above primary school	Low-income families	High-income families
Internet use	0.092^***^ (2.971)	0.073 (1.505)	0.050 (0.885)	0.084^***^ (2.879)	0.070^**^ (2.330)	0.078 (1.484)
Control variables	Yes	Yes	Yes	Yes	Yes	Yes
Province	Yes	Yes	Yes	Yes	Yes	Yes
Year	Yes	Yes	Yes	Yes	Yes	Yes
Observations	5729	2264	2366	5627	6382	1611

Values in parentheses are t-values (robust standard errors). ** and *** indicate p< 0.05 and p< 0.01, respectively.

## 5 Discussion

Research on Internet use and adolescent health is mainly focused on developed countries, and there is insufficient evidence from developing countries. Therefore, this paper uses the CFPS data from 2010, 2014, 2016, 2018, and 2020 to explore the impact of Internet use on the health of Chinese adolescents.

First, our study showed that the use of the Internet by adolescents was likely to damage their health, which is similar to previous studies in some developed countries (58–60). Second, by investigating potential mechanisms, we find that Internet use negatively impacts physical and mental health by increasing parent-child conflict and decreasing academic performance and self-efficacy, which is in line with the proposed hypothesis. This emphasizes the importance of families and schools in preventing adolescents from harming their health by using the Internet (61).

Finally, Heterogeneity analysis showed that Internet use hurt the physical and mental health of non-boarding students, but had no impact on the physical and mental health of boarding students. In boarding schools, the boarding students’ clothing, food, housing, and transportation are strictly managed. They cannot use mobile phones or computers and other Internet devices at will. They have fewer opportunities to use the Internet, so the possibility of being infringed by the Internet is lower.

China is still a division of labor pattern of “men in charge of the outside world and women in charge of the inside” (62). Fathers are more likely to work outside the home, and the responsibility of caring for adolescents is mainly borne by mothers. The education level of mothers has an important impact on the health of adolescents. Adolescents whose mothers’ education background is primary school or below are more likely to damage their self-rated health by using the Internet. This may be because mothers with low education are prone to adopt negative ways of discipline and are unable to make reasonable supervision of Adolescents’ use of the Internet (63). Adolescents whose mothers are educated above primary school are facing greater psychological pressure when using the Internet. Considering the influence of China’s culture of “looking forward to children’s success”, mothers with higher education have higher expectations for adolescents, and adolescents are forced to use the Internet to study and participate in interest classes, which aggravates their psychological pressure.

Adolescents from high-income families live in areas with higher Internet penetration and have sufficient conditions to use mobile phones, computers, and other Internet devices. They are more prone to being sedentary, staring at the screen for a long time, and other phenomena. Their self-rated health is more vulnerable to internet infringement. Contrary to the self-rated health results, the use of the Internet by adolescents from low-income families damaged their mental health, while the impact of adolescents from high-income families was not significant. According to the information gap theory, when people realize that there is a gap between what they know and what they want to know, they will be curious (64). Adolescents from low-income families have less access to the Internet, are more curious about the content on the Internet, are easily attracted by bad information, and their mental health is damaged.

This study had some limitations. First, due to data limitations, this study cannot further explore the impact of different Internet use purposes on adolescent health. Second, the youth samples used in this paper are between 10 and 15 years old, which may not fully reflect the impact of Internet use on the health of adolescents under 18 years old. Third, due to data limitations, we cannot include all variables that affect adolescents’ Internet use and physical and mental health.

## 6 Conclusion

This study empirically explored the impact of Internet use on the physical and mental health of adolescents in China. The results demonstrated that adolescents’ Internet use negatively affected their physical and mental health. Adolescents’ Internet use increased parent-child conflicts and reduced academic performance and self-efficacy. The negative effects of Internet use on adolescents’ physical and mental health are heterogeneous in boarding, mother’s education level, and family per capita income. The research remained robust after a series of tests.

These findings shed some light on the prevention and intervention of the damage of the Internet to the health of adolescents and offer a reference for the improvement of the protection policies for the use of the Internet by adolescents. Family and school are important links in preventing and intervening in the harm of the Internet to the health of adolescents. Therefore, parents should set reasonable online time and rules for adolescents, understand and guide their Internet activities, and help them develop good Internet use habits. Furthermore, parents should maintain good relationships and communication with adolescents, provide psychological support, and help them cope with the negative effects of the Internet. Schools should incorporate Internet security courses into the curriculum and focus on reducing the academic pressure on adolescents and cultivating their academic self-efficacy through relevant courses to improve their resistance to the negative impact of the Internet. Moreover, the government should enact relevant laws and policies to regulate the behavior of Internet content providers, accelerate the implementation of sound real-name and rating system policies, strengthen Internet management, and implement measures to protect minors.

## Data availability statement

Publicly available datasets were analyzed in this study. This data can be found here: https://doi.org/10.18170/DVN/45LCSO.

## Ethics statement

The studies involving humans were approved by the Biomedical Ethics Committee of Peking University (IRB00001052-14010). The studies were conducted in accordance with the local legislation and institutional requirements. Written informed consent for participation in this study was provided by the participants’ legal guardians/next of kin.

## Author contributions

ND: Conceptualization, Data curation, Methodology, Supervision, Writing – review & editing. LL: Conceptualization, Data curation, Formal analysis, Methodology, Writing – original draft, Writing – review & editing. LZ: Methodology, Writing – review & editing. SY: Project administration, Resources, Supervision, Writing – review & editing.

## Footnotes

1 Available from: https://www.ohchr.org/zh/health/global-strategy-womens-childrens-and-adolescents-health-2016-2030.

2 Students under 18 years old in school, excluding those under 6 years old and non-student samples.

3 The children's database only includes adolescents under 16 years old.

4 K6 self-assessment standard: 1. almost every day; 2. often; 3. half the time; 4. some time; 5. never.

5 CES-D self-assessment standard: 1. almost no; 2. sometimes; 3. often; 4. most of the time.

6 The average value of other adolescents' Internet usage in the community calculated after excluding themselves.

7 Yes indicates that the fixed effect of the corresponding province and year has been controlled.

8 In order to save space, the control variable coefficient is no longer displayed.

9 The per capita income of families is divided according to the 80% quantile of the whole sample, and those higher than the 80% quantile are high-income families, otherwise they are low-income families.
